# Greater chronic morbidity is associated with greater fatigue in six countries: A case of evolutionary mismatch?

**DOI:** 10.1093/emph/eoac011

**Published:** 2022-04-11

**Authors:** Joshua M Schrock, Lawrence S Sugiyama, Nirmala Naidoo, Paul Kowal, J Josh Snodgrass

**Affiliations:** 1 Institute for Sexual and Gender Minority Health and Wellbeing, Northwestern University, 625 N Michigan Avenue, Suite 14, Chicago, IL 60611, USA; 2 Department of Anthropology, Northwestern University, 1810 Hinman Avenue, Evanston, IL 60208, USA; 3 Department of Anthropology, 1218 University of Oregon, 308 Condon Hall, Eugene, OR 97403, USA; 4 Study on global AGEing and adult health (SAGE), World Health Organization, Avenue Appia 20, Geneva 1211, Switzerland; 5 Centre for Women’s Health Research, University Drive, Callaghan, NSW 2308, Australia; 6 Center for Global Health, University of Oregon, 1585 E 13th Avenue, Eugene, OR 97403, USA

**Keywords:** chronic diseases, mental health, aging, epidemiology

## Abstract

**Background and objectives:**

Human susceptibility to chronic non-communicable disease may be explained, in part, by mismatches between our evolved biology and contemporary environmental conditions. Disease-induced fatigue may function to reduce physical activity during acute infection, thereby making more energy available to mount an effective immune response. However, fatigue in the context of chronic disease may be maladaptive because long-term reductions in physical activity increase risks of disease progression and the acquisition of additional morbidities. Here, we test whether cumulative chronic morbidity is associated with subjective fatigue.

**Methodology:**

We constructed a cumulative chronic morbidity score using self-reported diagnoses and algorithm-based assessments, and a subjective fatigue score based on four questionnaire items using cross-sectional survey data from the Study on global AGEing and adult health, which features large samples of adults from six countries (China, Ghana, India, Mexico, Russia and South Africa).

**Results:**

In a mixed-effects linear model with participants nested in countries (*N* = 32 455), greater cumulative chronic morbidity is associated with greater subjective fatigue (*β* = 0.34, SE = 0.005, *P* < 2e−16). This association replicates within each country and is robust to adjustment for key sociodemographic and physical covariates (sex, age, household wealth, physical function score, habitual physical activity, BMI and BMI^2^).

**Conclusions and implications:**

Fatigue is a common but perhaps maladaptive neuropsychological response to chronic morbidity. Disease-induced fatigue may mediate a self-perpetuating cycle, in which chronic morbidity reduces physical activity, and less physical activity increases cumulative chronic morbidity. Longitudinal research is needed to test whether chronic morbidity, fatigue and physical activity form a cyclical feedback loop.

**Lay Summary:** Fatigue during acute illness may promote recovery, but persistent fatigue in the context of chronic disease may make matters worse. We present evidence from six countries that more chronic disease is associated with more fatigue. This fatigue may reduce physical activity, which increases risks of acquiring additional chronic health problems.

## BACKGROUND AND OBJECTIVES

Chronic non-communicable diseases (e.g. heart diseases, diabetes and cancer) now account for most of the global burden of death and disability [1, 2]. Epidemics of chronic disease tend to emerge in environments where extrinsic mortality rates are relatively low and obesity rates are relatively high [3].

These chronic non-communicable diseases were likely rare throughout the vast majority of our evolutionary history [4]. Obesity-related chronic disease risk factors are rare in contemporary hunter-gatherers and other minimally market-integrated subsistence societies [5, 6]. Infectious diseases, on the other hand, account for 20–85% of deaths in contemporary and ethnographically known hunter-gatherer groups [7–12]. The rise of agriculture (starting ∼10–15 000 BP) brought about new sources of infectious disease risk, with dense population centers and proximity to domesticated animals [13].

Despite periodic pandemics (e.g. COVID-19, influenza and HIV/AIDS), global infectious disease mortality rates have declined in the past 100–200 years [14] and rates of obesity have increased [15]. In many countries, obesity was once associated with high socioeconomic status but is now prevalent across social strata and disproportionately impacts those of low socioeconomic status [16, 17].

Reductions in global infectious disease mortality have been accompanied by an increase in life expectancy at birth, along with a dramatic increase chronic non-communicable disease [1–3]. Along with changes in diet and reductions in subsistence-related physical activity, longer average lifespans have led to a higher prevalence of aging-related chronic conditions (e.g. cardiometabolic diseases and cancers) [1–3]. Mortality from aging-related diseases was relatively rare for most of human evolutionary history, in part because fewer individuals lived long enough to die of these aging-related diseases [3]. In contrast, infectious disease has been a persistent cause of morbidity and mortality across the lifespan for most of our evolutionary history [7–12]. Our somatic maintenance systems may therefore be poorly adapted to prevent the kinds of morbidity and mortality that are most common in aging populations that consume highly processed, calorie-dense diets and exhibit low levels of physical activity [4]. In populations that have experienced evolutionarily novel increases in the average life expectancy, there is considerable variability in healthy aging [18]. Some individuals experience multiple decades of disability-free life in older adulthood, while other individuals experience rapid decline in functional status with age. Research is needed to identify processes across the lifecourse that explain this variation in healthy aging.

Given the rapid, recent rise of chronic non-communicable diseases as a major source of morbidity and mortality, our evolved brains and bodies may be poorly equipped to deal with these diseases [19]. Much of our susceptibility to chronic disease may be explained by mismatches between our evolved biology and contemporary environmental conditions [4].

For example, humans have a propensity to generate large fat reserves when it is nutritionally feasible [20]. In the environments typical of our evolutionary history, the capacity to form large fat deposits provided a mechanism to maintain a stable energy supply for funding the high metabolic costs of maintaining our large brains, supporting multiple dependent offspring, foraging and lactating during extended periods of negative energy balance [21, 22]. In contemporary environments characterized by calorie-dense foods and sedentary lifestyles, our propensity for adiposity makes us vulnerable to obesity.

Along the same lines, human immune systems seem to be mismatched with contemporary environments that are low in microbial diversity and feature low rates of exposure to infectious pathogens that were common for most of our evolutionary history (e.g. parasitic worms) [23, 24]. This lack of exposure to our long-time microbial and macroparasitic co-evolutionary companions influences the development of our immune systems, making us vulnerable to allergies, autoimmune diseases and inflammation-related disorders [24, 25].

In this article, we consider another feature of our evolved biology that appears to be mismatched with contemporary environments—disease-induced fatigue. Multiple lines of evidence suggest that disease-induced fatigue evolved to promote host survival during acute infection. But like chronic inflammation, fatigue may be counterproductive when it is deployed chronically in response to chronic non-communicable diseases.

Like other vertebrates, humans exhibit a typical neuropsychological response to internal immunological cues of infection or somatic damage [26, 27]. This response includes increased lethargy, social withdrawal, reduced appetite and increased pain sensitivity [26]. Animal behaviorists and psychoneuroimmunologists refer to these changes as sickness behavior [26, 28]. These changes are hypothesized to be adaptive adjustments that help organisms fight acute infection and recover from acute somatic damage [26, 27].

Depending on various contextual cues, sickness behavior includes: (i) increased fatigue to reduce physical activity, thereby making more energy available for the immune system; (ii) increased sensitivity to nausea and pain to reduce the risk of acquiring additional infections or injuries that would compound the immune system’s workload; (iii) changes in temperature perception to promote thermoregulatory behaviors that reduce the cost of maintaining or increasing body temperature; (iv) changes in appetite to promote consumption behaviors (or lack thereof) that support the fight against infection; and (v) detectable changes in facial expressions, body language and social behavior that signal to our social allies that we need help and support [29].

Acute infection, injury and chronic degenerative disease are all associated with increases in pro-inflammatory immune activity [30, 31]. Studies with human participants and animal models demonstrate that experimentally induced increases in pro-inflammatory cytokine production can initiate the psychological and behavioral changes characteristic of sickness behavior [26, 27].

During acute infection or injury, increased fatigue reduces physical activity, thereby making more energy available to mount an effective immune response [29, 32, 33]. During chronic disease, however, physical inactivity is an important causal factor driving disease progression and the acquisition of additional morbidities [34, 35]. In some cases, physical exercise can even reverse chronic non-communicable diseases, such as type 2 diabetes, though the doses of physical activity needed to reverse type 2 diabetes are much higher than the doses of physical exercise typically prescribed [36].

During acute infection, systemic inflammation is one of the main mechanistic cues that activates the neural mechanisms that regulate sickness behavior [29]. Systemic inflammation is often also elevated in people with chronic diseases, and some cases of chronic disease appear to induce a chronic version of sickness behavior [37, 38]. Along the same lines, greater systemic inflammation has been linked to greater risks for chronic fatigue and depression [39, 40]. Physical activity reduces inflammation through the action of myokines produced by active skeletal muscle [41]. Thus, physical inactivity can further exacerbate inflammation stemming from chronic diseases by failing to activate a major regulatory pathway that normally down-regulates inflammation. Thus, chronic morbidity may initiate a vicious self-perpetuating cycle, in which increased chronic morbidity triggers increased chronic fatigue, and greater chronic fatigue leads to even greater chronic morbidity by reducing levels of physical activity.

Besides inflammation, there are other mechanisms that may contribute to fatigue during chronic disease [37, 42]. For example, disrupted insulin regulation in the context of diabetes may interfere with one’s ability to mobilize metabolic resources, thereby inducing fatigue. Reduced oxygen supply may contribute to fatigue in people with chronic lung disease. The higher energy costs of movement may increase fatigue for people with high levels of adiposity. Cardiovascular damage may increase fatigue by limiting one’s ability to mount sufficient cardiac output. The discomfort of movement may exacerbate fatigue in arthritis. Recent research has demonstrated that total energy expenditure progressively declines in humans after the age of 60 in contemporary populations, so some cases of apparent chronic disease-related fatigue may simply reflect age-related declines in total energy expenditure [43]. For most of our evolutionary history, fatigue may have been an adaptive response when experiencing physiological cues of infection, metabolic depletion, tissue damage, hypoxia or prolonged pain [29]. In the context of chronic aging-related diseases, fatigue may be a maladaptive response that exacerbates the underlying chronic condition by reducing physical activity.

This feedback loop connecting chronic disease, fatigue and physical activity may play a role in generating the global pandemic of chronic non-communicable disease. One prediction arising from this model is that greater cumulative chronic morbidity is associated with greater subjective fatigue. We test this prediction using large cross-sectional samples of adults from six culturally distinct countries.

## METHODOLOGY

We used data from the World Health Organization’s (WHO) Study on global AGEing and adult health (SAGE), Wave 1, which collected cross-sectional data on aging-related health among adults in six middle-income countries (China, Ghana, India, Mexico, Russia and South Africa). In our analyses, we include all adults (ages 18+) from the Wave 1 dataset who have complete data for all variables included in our statistical models. Wave 1 data collection began in 2007 and concluded in 2010. Data collection protocols for SAGE are described in detail elsewhere [44]. All SAGE study protocols were approved by WHO’s Research Ethics Review Committee and by the ethical review organization with jurisdiction in each country. Written informed consent was obtained from all participants.

### Cumulative chronic morbidity

We created a cumulative chronic morbidity variable by summing the number of chronic conditions reported by each participant. Data were available on seven chronic conditions: arthritis, stroke, angina, diabetes, chronic lung disease, asthma and hypertension. For arthritis, angina, chronic lung disease and asthma, we followed previously published protocols for coding each condition as either present or absent [45]. A participant was coded as having the condition if they reported having been diagnosed with it, or if their responses to a symptom-based diagnostic algorithm indicated that they had the condition (for angina and stroke). Participants were coded as having hypertension if they reported ever having been diagnosed with it or if their measured resting blood pressure (average of three trials, except for the Mexico data, where an average of the two available trials was used) was greater than systolic =140 mmHg or diastolic =90 mmHg. Participants were coded as having diabetes if they reported having been diagnosed with it. Summing the number of chronic conditions for each individual generated a count variable ranging from 0 to 7.

### Subjective fatigue

We calculated a subjective fatigue score by summing responses to four questions. Question 1 asked, ‘Overall in the last 30 days, how much difficulty did you have in vigorous activities (vigorous activities require hard physical effort and cause large increases in breathing or heart rate)?’ Question 2 asked, ‘Overall in the last 30 days, how much of a problem did you have due to not feeling rested or refreshed during the day?’ Question 3 asked, ‘Do you have enough energy for everyday life?’ Question 4 asked, ‘During the last 12 months, have you had a period lasting several days when you have been feeling your energy decreased or that you are tired all the time?’ Questions 1–3 were answered on a 5-point ordinal scale, with higher values indicating greater subjective fatigue. Question 4 was a yes-or-no question, so we coded the response ‘no’ as 1 and the response ‘yes’ as 5 so that this item would have an influence on the subjective fatigue score equivalent to the other three items. The resulting subjective fatigue score ranged from 4 to 20.

### Covariates

Age was reported as chronological age in years. Sex was reported as male or female. Household wealth was a composite measure based on possession of durable goods, dwelling characteristics and access to services [44]. Responses to 21 items were coded as ‘1’ (denoting possession or access to the item) or ‘0’ (denoting a lack of possession or access to the item). In a reshaped dataset, each response item was then treated as a separate observation for wealth in a pure random effects model, which produced indicator-specific thresholds for a latent wealth scale. Households were then arranged into a country-specific asset ladder using an empirical Bayes postestimation method. The value of this asset ladder was assigned to individuals as their wealth score.

A physical function score was calculated by combining performance on two different timed walk tasks and grip strength for each hand. One timed walk task involved walking 4 m at a normal pace. The other timed walk task involved walking 4 m as quickly as possible. Grip strength was measured using a dynamometer with two trials for each hand. We averaged the two trials in each hand to create an average for the dominant and non-dominant hands. If no dominant hand was reported, the right hand was coded as dominant. Normal walk time and rapid walk time were each standardized by stature separately for men and women. Dominant grip strength and non-dominant grip strength were each standardized by body weight separately for men and women. These standardized scores were combined to generate an overall physical function score. To remove implausible values and extreme outliers, we dropped cases for which the physical performance score was more than four standard deviations from the mean.

Following the Global Physical Activity Questionnaire analysis guide [46], we calculated a habitual physical activity variable that multiplies the minutes per week spent in each category of activity by the metabolic equivalent (MET) for that activity (the estimated ratio of total energy expenditure required to perform the task to the energy expenditure required for just resting). The resulting MET-minutes variable integrates both the quantity and intensity of an individual’s physical activity levels in a typical week.

Body mass index (BMI, kg/m^2^) was calculated from stature and weight measured using standard protocols.

### Statistical analysis

In analyses with all countries combined, we specified mixed-effects linear models with participants nested in countries (i.e. with a random effect for country) in the R package ‘lme4’ (version 1.1-19). In analyses that considered each country separately, we specified ordinary least squares multiple regression models in base R (version 3.5.0). We created plots using the R package ‘ggplot2’ (version 3.1.0).

In the first set of models, only cumulative chronic morbidity and an intercept term were included as predictors of subjective fatigue. In the second set of models, the sociodemographic covariates (age, sex and wealth) were included, in addition to the terms in the first set of models. In the third set of models, the physical covariates (physical function score, physical activity level, BMI and BMI^2^) were included, in addition to the terms in the second set of models.

Models with physical covariates included terms for both BMI and BMI^2^ because we expected BMI to have both linear effects (greater body mass represents greater energetic reserves and, therefore, less subjective fatigue) and curvilinear effects (those in the underweight and obese extremes of BMI are expected to have greater subjective fatigue).

All variables except age, sex and cumulative chronic conditions were standardized prior to analysis (mean =0, SD = 1). Physical activity levels (MET-minutes) and subjective fatigue scores were positively skewed and were natural log-transformed prior to standardization.

## RESULTS

The distribution of age, sex and cumulative chronic morbidity by country is presented in Table 1.

**Table 1.** Sample distribution of sex, age, cumulative chronic disease burden and subjective fatigue score by country

	China	Ghana	India	Mexico	Russia	South Africa	Total
	(*n* = 12 319)	(*n* = 3792)	(*n* = 9271)	(*n* = 1888)	(*n* = 2387)	(*n* = 2798)	(*N* = 32 455)
Female, *n* (%)	6566 (53.3)	1693 (44.6)	5603 (60.4)	1150 (60.9)	1499 (62.8)	1564 (55.9)	18075 (55.7)
Age, μ (SD)	60 (11.7)	58.7 (14)	49.1 (16.4)	61.7 (13.9)	59.9 (12.8)	59.8 (12.2)	56.8 (14.6)
Number of chronic conditions, *n* (%)							
0	3548 (28.8)	1155 (30.5)	4353 (47)	467 (24.7)	533 (22.3)	490 (17.5)	10546 (32.5)
1	5093 (41.3)	1762 (46.5)	2909 (31.4)	796 (42.2)	621 (26)	1465 (52.4)	12646 (39)
2	2467 (20)	695 (18.3)	1269 (13.7)	423 (22.4)	625 (26.2)	574 (20.5)	6053 (18.7)
3	834 (6.8)	149 (3.9)	494 (5.3)	142 (7.5)	348 (14.6)	185 (6.6)	2152 (6.6)
4	280 (2.3)	23 (0.6)	189 (2)	43 (2.3)	171 (7.2)	60 (2.1)	766 (2.4)
5	83 (0.7)	—	49 (0.5)	14 (0.7)	74 (3.1)	20 (0.7)	244 (0.8)
6	12 (0.1)	—	6 (0.06)	—	14 (0.6)	—	42 (0.1)
7	—	—	—	—	—	—	6 (0.02)
Subjective fatigue scores by number of chronic conditions, median (25th percentile, 75th percentile)							
0	−0.5 (−1.0, −0.1)	−0.1 (−1.0, 0.8)	−0.1 (−1.0, 0.5)	−0.5 (−1.0, 0.2)	−0.5 (−1.0, 0.2)	−1.0 (−1.0, −0.1)	−0.5 (−1.0, 0.2)
1	−0.5 (−1.0, 0.2)	0.2 (−0.5, 0.8)	0.2 (−0.5, 1.0)	−0.1 (−1.0, 0.5)	−0.1 (−1.0, 0.8)	−0.5 (−1.0, 0.2)	−0.1 (−1.0, 0.5)
2	−0.1 (−0.5, 0.5)	0.8 (0.2, 1.3)	1.0 (0.2, 1.5)	0.2 (−0.5, 0.8)	0.5 (−0.1, 1.3)	0.2 (−0.5, 0.5)	0.2 (−0.5, 1.0)
3	0.2 (−0.1, 0.8)	1.0 (0.5, 1.5)	1.3 (0.5, 1.8)	0.8 (0.2, 1.4)	1.0 (0.2, 1.7)	0.5 (−0.1, 1.0)	0.8 (−0.1, 1.3)
4	0.5 (−0.1, 1.3)	1.0 (0.8, 1.7)	1.5 (1.0, 2.0)	1.0 (0.0, 1.5)	1.3 (0.5, 1.8)	0.8 (0.1, 1.3)	1.0 (0.2, 1.7)
5	0.8 (0.0, 1.5)	—	1.7 (1.3, 2.0)	1.5 (1.0, 1.9)	1.5 (0.8, 1.8)	1.4 (0.5, 1.7)	1.3 (0.5, 1.8)
6	1.3 (0.9, 1.7)	—	2.1 (2.0, 2.2)	—	1.7 (0.9, 1.8)	—	1.5 (0.8, 1.8)
7	—	—	—	—	—	—	2.1 (1.5, 2.2)

—Cells with fewer than five participants censored to protect anonymity.

### All countries combined

Model 1 included fixed effects for cumulative chronic morbidity, a random effect for country, and the model intercept as predictors of subjective fatigue. Greater cumulative chronic morbidity was associated with greater subjective fatigue (*β* = 0.34, SE = 0.005, *P* < 2e-16). Model 2 contained all the terms in Model 1 as well as fixed effects for age, sex and wealth. Greater cumulative chronic morbidity was also associated with greater subjective fatigue in Model 2 (*β* = 0.25, SE = 0.005, *P* < 2e-16). Model 3 contained all the terms in Model 2, as well as fixed effects for physical function score, physical activity levels, BMI and BMI^2^. Greater cumulative chronic morbidity was also associated with greater subjective fatigue in Model 3 (*β* = 0.25, SE = 0.005, *P* < 2e-16).

In the mixed models combining data from all countries, the fully adjusted model (Model 3) exhibited the highest quality as indexed by both Akaike information criterion (AIC) and Bayes information criterion (BIC).

### Within-country analyses

Analyses stratified by country included the same set fixed effect terms as the pooled analyses with all countries combined. In Model 1, greater cumulative chronic morbidity was associated with greater subjective fatigue in all countries: China (*β* = 0.29, SE = 0.007, *P* < 2e-16), Ghana (*β* = 0.315, SE = 0.017, *P* < 2e-16), India (*β* = 0.392, SE = 0.01, *P* < 2e-16), Mexico (*β* = 0.323, SE = 0.021, *P* < 2e-16), Russia (*β* = 0.394, SE = 0.014, *P* < 2e-16) and South Africa (*β* = 0.0.345, SE = 0.016, *P* < 2e-16).

In Model 2, greater cumulative chronic morbidity was also associated with greater subjective fatigue in all countries: China (*β* = 0.222, SE = 0.007, *P* < 2e-16), Ghana (*β* = 0.217, SE = 0.015, *P* < 2e−16), India (*β* = 0.262, SE = 0.009, *P* < 2e−16), Mexico (*β* = 0.27, SE = 0.021, *P* < 2e−16), Russia (*β* = 0.323, SE = 0.014, *P* < 2e−16) and South Africa (*β* = 0.315, SE = 0.016, *P* < 2e−16).

The same was true in Model 3: China (*β* = 0.228, SE = 0.007, *P* < 2e−16), Ghana (*β* = 0.207, SE = 0.015, *P* < 2e−16), India (*β* = 0.262, SE = 0.009, *P* < 2e−16), Mexico (*β* = 0.271, SE = 0.021, *P* < 2e−16), Russia (*β* = 0.31, SE = 0.015, *P* < 2e−16) and South Africa (*β* = 0.299, SE = 0.016, *P* < 2e−16).

In within-country models, the fully adjusted model (Model 3) exhibited the highest quality in all countries as indexed by AIC. In all countries except the Russian Federation, the fully adjusted model (Model 3) exhibited the highest quality as indexed by BIC. In the Russian Federation, the model with only the intercept, cumulative chronic morbidity and sociodemographic covariates (Model 2) exhibited the highest quality as indexed by BIC.

**Figure 1.**
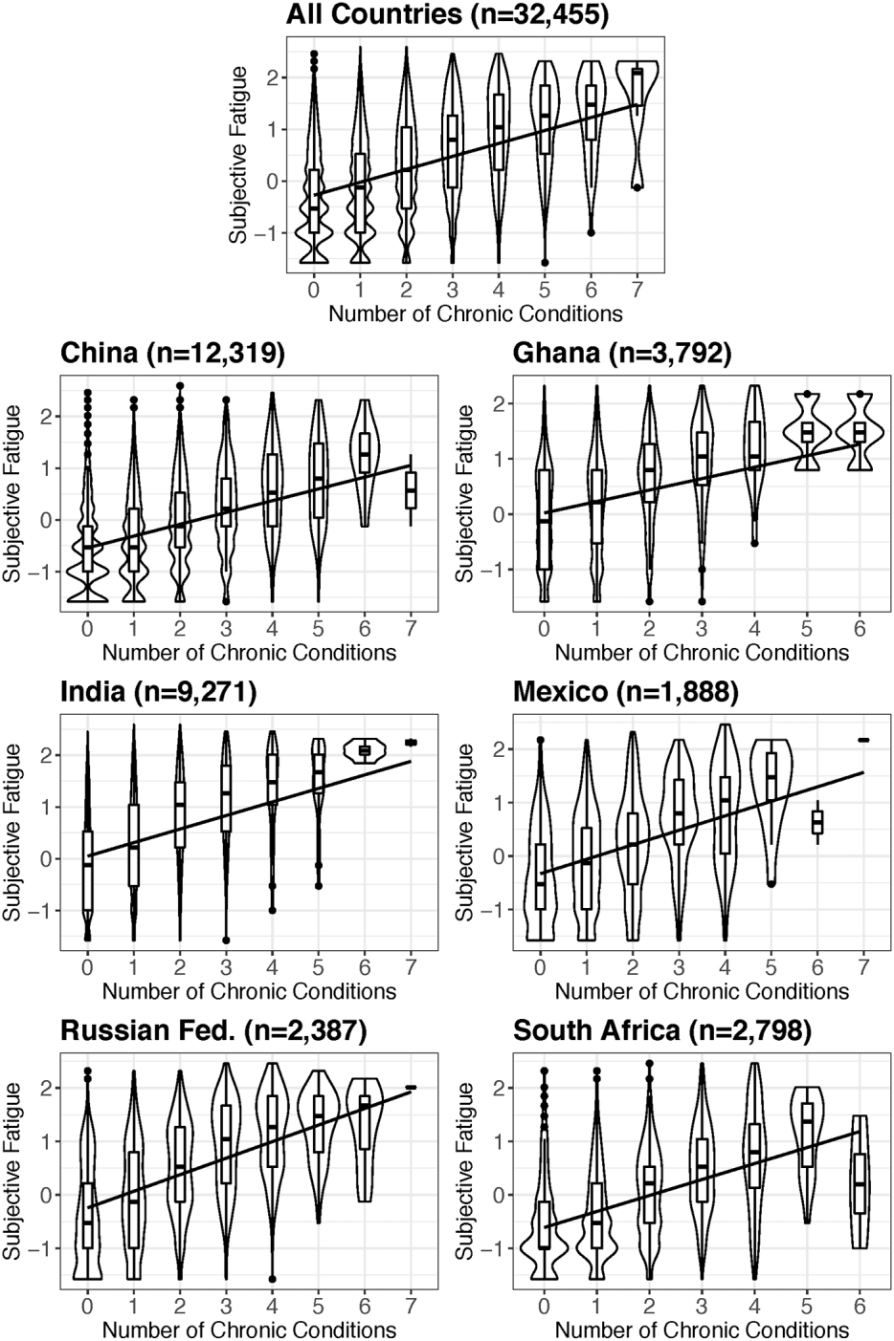
Cumulative chronic morbidity and subjective fatigue scores by country. The boxplot within each violin plot represents the interquartile range of subjective fatigue scores for each value of the chronic disease count, with the thick horizontal line in the middle of the boxplot indicating the median value of subjective fatigue scores for that category. The smoothed kernel density plot that surrounds each boxplot represents the distribution of subjective fatigue scores for each value of the chronic disease count. Wider regions of the smoothed kernel density plot indicate greater frequencies. In the dataset with all countries combined, each additional chronic condition is associated with an increase in subjective fatigue score. These patterns are broadly similar across countries. The plotted line represents the marginal effects from a fully adjusted regression model. Subjective fatigue scores were natural log-transformed and standardized (mean =0, SD = 1) prior to plotting

In Table 2, we present coefficients, standard errors and *P*-values for all models. In Fig. 1, we use violin plots and plotted lines representing marginal effects to visualize the relationship between cumulative chronic morbidity and subjective fatigue by country.

**Table 2.** Linear models with subjective fatigue scores as the dependent variable

	All countries (*N* = 32 455)		
	Model 1	Model 2	Model 3
Intercept			
*Β*	−0.36	−1.62	−1.47
SE	0.11	0.17	0.15
*P*	0.0225	0.0002	0.0001
Chronic conditions			
*Β*	0.34	0.25	0.25
SE	0.005	0.005	0.005
*P*	<2e−16	<2e−16	<2e−16
Sex			
*Β*		0.23	0.23
SE		0.009	0.009
*P*		<2e−16	<2e−16
Age			
*Β*		0.021	0.019
SE		0.0004	0.0004
*P*		<2e−16	<2e−16
Wealth			
*Β*		−0.204	−0.186
SE		0.006	0.006
*P*		<2e−16	<2e−16
Physical function			
*Β*			−0.083
SE			0.005
*P*			<2e−16
Physical activity			
*Β*			−0.018
SE			0.005
*P*			0.0003
BMI			
*Β*			−0.09
SE			0.006
*P*			<2e−16
BMI^2^			
*Β*			0.061
SE			0.006
*P*			<2e−16
Country (random effect)			
Intercept			
Variance	0.07493	0.1616	0.1234
SD	0.2737	0.402	0.3513
Residual			
Variance	0.80514	0.6893	0.6791
SD	0.8973	0.8302	0.8241
BIC	85 155.86	80 177.15	79 763.3
AIC	85 122.31	80 118.43	79 671.04
Log likelihood	−42 557.2	−40 052.2	−39 824.5

	China (*n* = 12 319)		
	Model 1	Model 2	Model 3
Intercept			
*β*	−0.617	−1.70	−1.53
SE	0.012	0.039	0.042
*P*	<2e−16	<2e−16	<2e−16
Chronic conditions			
*β*	0.29	0.222	0.228
SE	0.007	0.007	0.007
*P*	<2e−16	<2e−16	<2e−16
Sex			
*β*		0.181	0.181
SE		0.014	0.014
*P*		<2e−16	<2e−16
Age			
*β*		0.015	0.013
SE		0.0007	0.0007
*P*		<2e−16	<2e−16
Wealth			
*β*		−0.288	−0.278
SE		0.009	0.009
*P*		<2e−16	<2e−16
Physical function			
*β*			−0.076
SE			0.009
*P*			<2e−16
Physical activity			
*β*			−0.012
SE			0.007
*P*			0.103
BMI			
*β*			−0.106
SE			0.012
*P*			<2e−16
BMI^2^			
*β*			0.056
SE			0.013
*P*			0.00001
BIC	31 112.68	29 312.67	29 194.04
AIC	31 090.42	29 268.16	29 119.85
Log likelihood	−15 542.2	−14 628.1	−14 549.9
Adjusted *r*^2^	0.11	0.24	0.25

	Ghana (*n* = 3792)		
	Model 1	Model 2	Model 3
	
	Model 1	Model 2	Model 3
Intercept			
*β*	−0.084	−1.78	−1.68
SE	0.022	0.056	0.603
*P*	0.0002	<2e−16	<2e−16
Chronic conditions			
*β*	0.315	0.217	0.207
SE	0.017	0.015	0.015
*P*	<2e−16	<2e−16	<2e−16
Sex			
*β*		0.286	0.323
SE		0.026	0.026
*P*		<2e−16	<2e−16
Age			
*β*		0.029	0.026
SE		0.0009	0.001
*P*		<2e−16	<2e−16
Wealth			
*β*		−0.148	−0.123
SE		0.016	0.017
*P*		<2e−16	1.38e−13
Physical function			
*β*			−0.093
SE			0.013
*P*			8.86e−13
Physical activity			
*β*			0.035
SE			0.014
*P*			0.012
BMI			
*β*			−0.073
SE			0.017
*P*			0.00001
BMI^2^			
*β*			0.045
SE			0.018
*P*			0.0133
BIC	9983.667	8945.119	8904.073
AIC	9964.946	8907.675	8841.666
Log likelihood	−4979.47	−4447.84	−4410.83
Adjusted *r*^2^	0.08	0.31	0.32

	India (*n *= 9271)		
	Model 1	Model 2	Model 3
Intercept			
*β*	−0.063	−1.32	−1.27
SE	0.013	0.035	0.04
*P*	0.000001	<2e−16	<2e−16
Chronic conditions			
*β*	0.392	0.262	0.262
SE	0.01	0.009	0.009
*P*	<2e−16	<2e−16	<2e−16
Sex			
*β*		0.337	0.326
SE		0.019	0.019
*P*		<2e−16	<2e−16
Age			
*β*		0.028	0.025
SE		0.0006	0.0007
*P*		<2e−16	<2e−16
Wealth			
*β*		−0.226	−0.196
SE		0.01	0.011
*P*		<2e−16	<2e−16
Physical function			
*β*			−0.117
SE			0.015
*P*			5.90e−15
Physical activity			
*β*			−0.006
SE			0.011
*P*			0.58
BMI			
*β*			−0.081
SE			0.012
*P*			3.08e−11
BMI^2^			
*β*			0.063
SE			0.015
*P*			0.00001
BIC	25 515.64	23 328.67	23 231.41
AIC	25 494.24	23 285.86	23 160.07
Log likelihood	−12 744.1	−11 636.9	−11 570.03
Adjusted *r*^2^	0.16	0.33	0.34

	Mexico (*n* = 1888)		
	Model 1	Model 2	Model 3
Intercept			
*β*	−0.396	−1.23	−0.987
SE	0.033	0.1	0.113
*P*	<2e−16	<2e−16	<2e−16
Chronic conditions			
*β*	0.323	0.27	0.271
SE	0.021	0.021	0.021
*P*	<2e−16	<2e−16	<2e−16
Sex			
*β*		0.337	0.319
SE		0.043	0.044
*P*		8.67e−15	3.72e−13
Age			
*β*		0.011	0.006
SE		0.002	0.002
*P*		2.06e−11	0.0004
Wealth			
*β*		−0.081	−0.072
SE		0.029	0.029
*P*		0.005	0.014
Physical function			
*β*			−0.114
SE			0.033
*P*			0.0006
Physical activity			
*β*			−0.08
SE			0.019
*P*			0.00004
BMI			
*β*			−0.051
SE			0.039
*P*			0.198
BMI^2^			
*β*			0.014
SE			0.03
*P*			0.649
BIC	5095.952	5008.227	5004.198
AIC	5079.322	4974.967	4948.765
Log likelihood	−2536.66	−2481.49	−2464.38
Adjusted *r*^2^	0.12	0.17	0.18

	Russian Federation (*n* = 2387)		
	Model 1	Model 2	Model 3
Intercept			
*β*	−0.387	−1.33	−1.1
SE	2.95E−02	8.93E−02	1.01E−01
*P*	<2e−16	<2e−16	<2e−16
Chronic conditions			
*β*	0.394	0.323	0.31
SE	0.014	0.014	0.015
*P*	<2e−16	<2e−16	<2e−16
Sex			
*β*		0.282	0.259
SE		0.038	0.038
*P*		8.12e−14	1.18e−11
Age			
*β*		0.014	0.011
SE		0.002	0.002
*P*		<2e−16	1.26e−10
Wealth			
*β*		−0.106	−0.088
SE		0.027	0.027
*P*		0.00008	0.001
Physical function			
*β*			−0.089
SE			0.018
*P*			0.000001
Physical activity			
*β*			−0.016
SE			0.023
*P*			0.502
BMI			
*β*			−0.017
SE			0.031
*P*			0.584
BMI^2^			
*β*			0.03
SE			0.022
*P*			0.177
BIC	6315.323	6168.611	6170.37
AIC	6297.99	6133.945	6112.592
Log likelihood	−3146	−3060.97	−3046.3
Adjusted *r*^2^	0.26	0.31	0.32

	South Africa (*n* = 2798)		
	Model 1	Model 2	Model 3
Intercept			
*β*	−0.669	−1.5	−1.28
SE	0.026	0.078	0.079
*P*	<2e−16	<2e−16	<2e−16
Chronic conditions			
*β*	0.345	0.315	0.299
SE	0.016	0.016	0.016
*P*	<2e−16	<2e−16	<2e−16
Sex			
*β*		0.127	0.153
SE		0.031	0.031
*P*		0.0004	8.07^−7^
Age			
*β*		0.014	0.011
SE		0.001	0.001
*P*		<2e−16	<2e−16
Wealth			
*β*		−0.094	−0.086
SE		0.016	0.016
*P*		1.20e−08	1.28e−07
Physical function			
*β*			−0.087
SE			0.009
*P*			<2e−16
Physical activity			
*β*			−0.075
SE			0.012
*P*			4.60e−10
BMI			
*β*			−0.097
SE			0.021
*P*			2.51e−06
BMI^2^			
*β*			0.05
SE			0.013
*P*			0.0001
BIC	6929.116	6780.346	6657.881
AIC	6911.306	6744.726	6598.514
Log likelihood	−3452.65	−3366.36	−3289.26
Adjusted *r*^2^	0.14	0.19	0.23

## DISCUSSION

We find that greater cumulative chronic morbidity is consistently associated with greater subjective fatigue. In the multilevel model including all participants, having four additional chronic conditions is associated with a full standard deviation increase in levels of subjective fatigue. This pattern is remarkably consistent across samples from six countries that are culturally and geographically distinct. Adding key physical variables to the model (physical function score, habitual physical activity, BMI and BMI^2^) does not substantially diminish the association between cumulative chronic morbidity and subjective fatigue in any model, which suggests that this association is not mediated by declines in physical capacity. Plotting subjective fatigue by number of chronic conditions reveals a dose–response pattern—each additional chronic condition is associated with a higher level of subjective fatigue. This pattern suggests that subjective fatigue is actually associated with cumulative chronic morbidity, not just with one or two of the chronic conditions aggregated in the variable.

In within-country analyses, the mean adjusted *r*^2^ in models with only cumulative chronic disease burden as the independent variable was 0.15 (range: 0.08–0.26), indicating that cumulative chronic disease burden alone explained about 15% of the variation in subjective fatigue scores. The mean adjusted *r*^2^ in the full model was 0.27 (range: 0.18–0.34). Cumulative chronic disease burden, along with other variables in the model, explained a substantial proportion of the variation in subjective fatigue scores. But there is also considerable remaining variation that remains unexplained in these models, reflecting the complex etiology of subjective fatigue.

Our findings suggest that subjective fatigue may be useful as a low-cost, non-invasive marker of cumulative pathology across a variety of physiological systems. Our findings dovetail with a previous study of the general UK population, which found that greater fatigue predicted higher hazards of all-cause and cardiovascular disease-related mortality, even after adjusting for a variety of potential confounders [47]. Along the same lines, a study of older US adults found that a single-item measure of fatigue at baseline (‘do you feel tired most of the time?’) predicted mortality rates 10 years later [48].

In this study, we utilize relatively low-resolution measures of cumulative chronic morbidity and subjective fatigue. Even so, we find that greater cumulative chronic disease burden exhibits consistent associations with greater subjective fatigue. Using measures that capture a wider range and finer grain of variation in these variables might yield even stronger associations between cumulative chronic morbidity and fatigue.

A limitation of this study is its reliance on self-report measures of physical activity and subjective fatigue. The limitations of self-reported physical activity are well documented—there is imprecise correspondence with objective measures and participants generally overestimate physical activity [49]. The limitations of self-reported fatigue are not precisely analogous to those of self-reported physical activity. For retrospectively self-reported physical activity, participants must attempt to recall and mentally aggregate behaviors across a specified timeframe. For subjective fatigue, the perception of fatigue is itself the target construct. Nonetheless, there are other ways of conceptualizing and measuring fatigue or fatigability, such as measuring the rate of decline in performance on standardized tasks [42]. Further research is needed to test whether our results replicate when using objective measures of physical activity and fatigue.

Another limitation of this study is that our measures of physical function were not comprehensive. Future studies should test whether our results replicate when controlling for more comprehensive indices of physical function, including measures of cardiorespiratory fitness, such as maximal oxygen consumption.

A third limitation of this article is that it does not identify the mechanisms linking chronic morbidity and fatigue. Given the analogous role of acute inflammation in generating fatigue during acute infection, inflammation is one possible mechanism linking chronic disease and fatigue [32]. However, fatigue in the context of chronic non-communicable diseases may have different mechanistic origins than fatigue during acute infection [42]. There are other possible physiological cues that could induce fatigue in the context of chronic non-communicable diseases, including reduced energy accessibility due to metabolic dysregulation, reduced oxygen availability, inability to mount sufficient cardiac output, tissue damage and increased discomfort when moving. Further research is needed to identify mechanisms linking chronic morbidity and fatigue.

**Figure 2.**
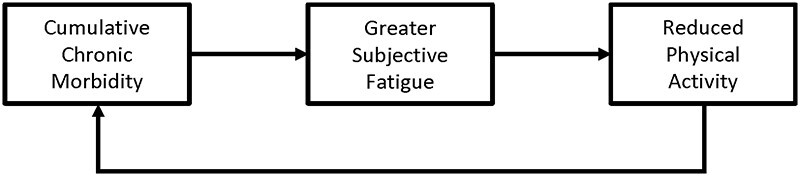
Hypothesized feedback loop linking cumulative chronic morbidity, subjective fatigue and physical activity

Our model proposes a cyclical relationship between chronic morbidity and fatigue—greater morbidity induces greater fatigue, and greater fatigue increases risks for additional morbidity by reducing long-term levels of physical activity (Fig. 2). A limitation of this study is that our cross-sectional study design cannot determine the temporal order of cumulative chronic morbidity and subjective fatigue. The cross-sectional associations, we observed in this study could be explained by chronic morbidity causing fatigue, fatigue causing chronic morbidity through reduced physical activity, or some combination of both. Longitudinal research is needed to determine whether increases in cumulative chronic morbidity predict subsequent increases in subjective fatigue. Clinical research is also needed to test whether interventions that reduce chronic disease-related fatigue are also successful in reducing subsequent morbidity and mortality.

There are specific features of many contemporary environments that may contribute to chronic disease-induced fatigue. Low microbial diversity, low rates of exposure to ancestrally common pathogens and obesity are all thought to play a role in the etiology of chronic inflammation [23, 25]. Experimental studies have demonstrated that inflammatory processes play a key mechanistic role in inducing disease-related changes in behavior and psychology, including increased fatigue [30]. Thus, chronic inflammation may initiate the self-reinforcing feedback cycle of declining physical activity and increasing chronic morbidity, even before the appearance of clinically identifiable chronic disease.

In contemporary medicine, individuals diagnosed with chronic non-communicable diseases are often prescribed exercise (discretionary physical activity undertaken for the sake of health and fitness) [22]. The literature has demonstrated that adherence to prescribed exercise is often low in people with chronic non-communicable diseases. Another potential issue is that fatigue may lead people with chronic diseases to inadvertently ‘compensate’ for bouts of exercise by reducing physical activity in other domains of life (e.g. work and recreation). Research is needed to evaluate the role of chronic disease-related fatigue in shaping adherence to prescribed exercise.

It is worth noting that we chose not to include depressive symptoms or self-reported diagnosis with a depressive disorder as a covariate in our statistical models because depression is likely a collider variable for cumulative chronic disease and persistent fatigue. Chronic physical disease predicts greater risks for depression [38], and persistent fatigue is a symptom that can contribute to classification as having a depressive disorder [31]. Thus, depression diagnoses may be causally ‘downstream’ from both cumulative chronic disease and persistent fatigue. Controlling for variables that are causally downstream from the independent and dependent variables introduces collider bias, which can produce misleading results [50]. Future studies aimed at investigating the interplay between cumulative chronic disease, fatigue and other symptoms of depression should utilize statistical approaches designed for simultaneously examining multiple outcome variables (e.g. regularized partial correlation networks and structural equation modeling).

## CONCLUSIONS AND IMPLICATIONS

In this article, we show that greater cumulative chronic morbidity is associated with greater subjective fatigue in six culturally diverse countries. Prior research has demonstrated that some parts of our evolved biology, such as our propensity for adiposity and our immune systems, are mismatched with contemporary environments where diets are calorie-dense, occupations are sedentary, microbial diversity is low and ancestrally common pathogens are rare. During acute infection, disease-induced fatigue may improve host survival by reducing physical activity, thereby making more energy available to mount an immune response. In the context of chronic non-communicable diseases, fatigue and reduced physical activity may increase risks of disease progression and acquisition of additional morbidities. Disease-induced fatigue may be another example of an evolved mechanism that is mismatched with many of the environments that humans currently inhabit.
